# Papillary Thyroid Carcinoma With Lymphoepithelial Features and Lacking Association With Epstein-Barr Virus (EBV): A Rare Case

**DOI:** 10.7759/cureus.55222

**Published:** 2024-02-29

**Authors:** Ahmed Bendari, Saroja Devi Geetha, Reham Al-Refai, Xuelin Zhong, Sunder Sham, Manju Harshan

**Affiliations:** 1 Department of Pathology, Lenox Hill Hospital, New York, USA; 2 Department of Pathology, Zucker School of Medicine, North Shore University Hospital/Long Island Jewish Medical Center, Northwell Health, Greenvale, USA; 3 Department of Pathology, Lenox Hill hospital, New York, USA

**Keywords:** lymphoepithelial features, lymphocytic thyroiditis, psammoma bodies, epstein-barr virus, papillary thyroid carcinoma with lymphoepithelial features

## Abstract

Papillary thyroid carcinoma (PTC) is the most common primary thyroid malignancy. PTC is diagnosed based on its hallmark nuclear characteristics, but a myriad of histological variants has been identified some of which can be diagnostically challenging due to its rarity and overlapping histomorphology with other entities. We report a rare variant of PTC with lymphoepithelial features which lacked association with Epstein-Barr Virus (EBV). In such cases, a thorough workup to rule out metastasis from other sites should be undertaken.

## Introduction

Papillary thyroid carcinoma (PTC) is the most common primary thyroid malignancy accounting for approximately 80% of cases [1,2]. Histologically, it is characterized by nuclear features like nuclear enlargement, nuclear overlapping, intranuclear grooves, chromatin clearing, and intranuclear cytoplasmic pseudoinclusions [3]. WHO (World Health Organization) classification 5th edition recognizes 13 subtypes of PTC which include tall cell, hobnail, columnar cell, diffuse sclerosing, solid, clear cell, spindle cell, and Warthin-like subtypes [4,5]. We report an unusual histologic pattern of PTC with lymphoepithelial features which lacked association with Epstein-Barr Virus (EBV).

This article was previously posted to Research Square preprint server on January 17, 2023.

## Case presentation

**Figure 1:**
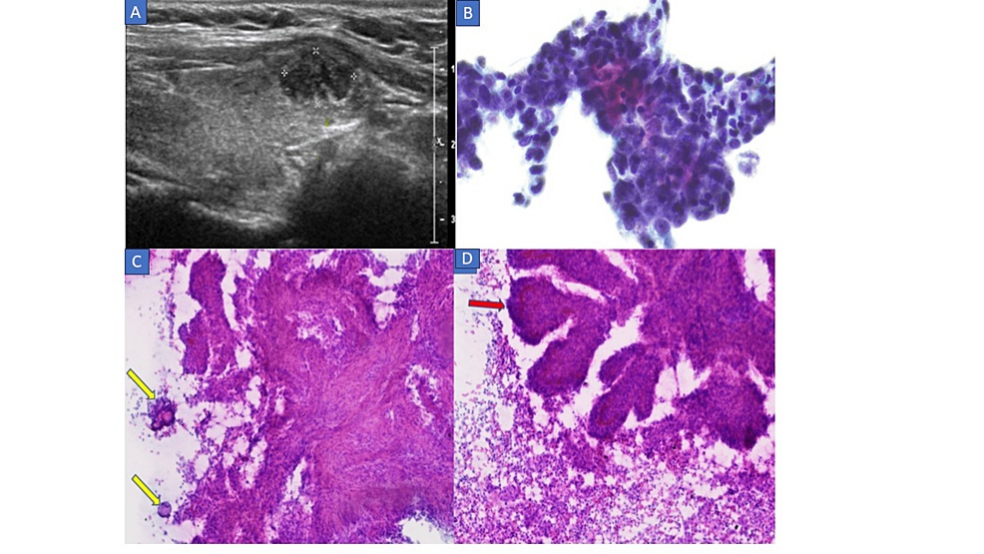
Thyroid U/S and FNAC A 1.5 cm hypoechoic solid nodule with ill-defined margins (A), FNAC (B, 20X) showing follicular cells with mild atypia, FNAC (C, 10X) showing psammoma bodies (yellow arrows) and (D, 10X) showing papillary architecture (red arrow) in the background of a mixed population of lymphocyte.

**Figure 2:**
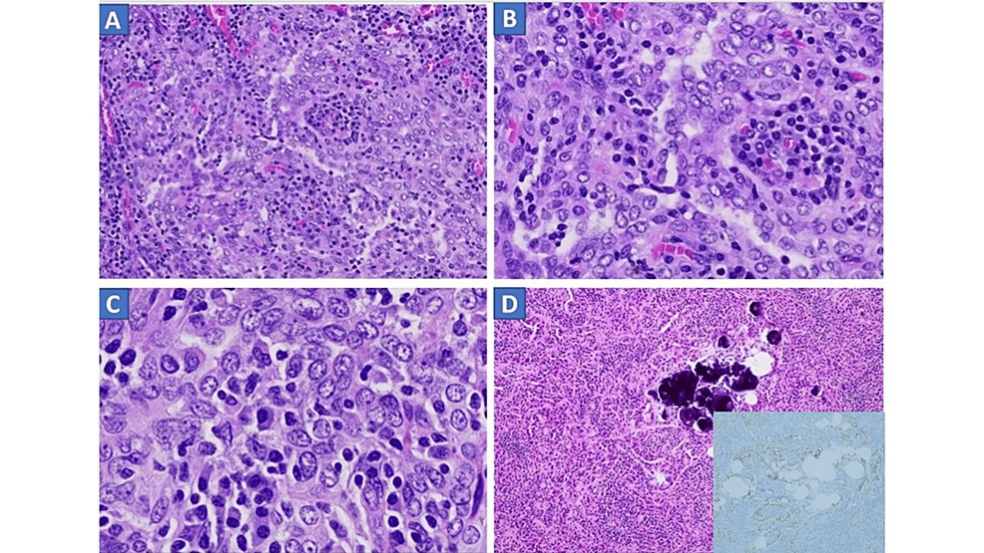
H&E examination Solid arborizing bands of oval to spindle cells with lymphocytic infiltration (A, 20X and B, 40X), tumor cells with nuclear membrane irregularities and intranuclear clearing (C, 60X), lymph node with metastasis and TTF-1 stain confirming metastasis (D, 20X).

A previously healthy 29-year-old female was first noted by her primary care physician to have hypercalcemia in 2019. Laboratory evaluation showed high levels of parathyroid hormone (PTH) (180.8 pg/ml) with low serum vitamin D (16.4 ng/ml). Thyroid hormones were within normal range and antithyroid antibodies were present. Thyroid ultrasound and Sestamibi scan showed a 1.4 cm parathyroid adenoma arising from the left inferior parathyroid gland and non-suspicious micro-nodularity in the thyroid. The patient had no family history of thyroid cancer, but her mother and maternal aunt had hypothyroidism. She underwent parathyroid adenoma resection. Her postoperative course was unremarkable until January 2022, when she noted a palpable nodule in the right thyroid lobe. Thyroid ultrasound showed a 1.5 cm hypoechoic solid nodule with ill-defined margins. Fine Needle Aspiration Cytology (FNAC) evaluation of the nodule showed hypercellularity with mild nuclear atypia of follicular cells and lymphocytes in the background (Bethesda category 3) (Figure 1B). The Afirma genomic sequencing classifier was reported as suspicious with a risk of malignancy ~ 50% and the Afirma expression atlas showed no variant or fusion. At three-month follow-up, the nodule had increased in size (1.85 cm), and repeat FNAC revealed follicular cells with nuclear enlargement, nuclear overlapping, focal three-dimensional clusters with papillary architecture, few psammoma bodies, and a mixed population of lymphocytes. The findings were suspicious for papillary thyroid carcinoma (Figure 1C, 1D). Thyroseq V3 genomic sequencing revealed gene expression alterations associated with thyroid cancer with a 60% risk for malignancy and no gene mutations or fusions. The patient underwent total thyroidectomy and paratracheal lymphadenectomy. Grossly the thyroid nodule was tan, firm, and well-circumscribed measuring 1.5 cm in size (Figure 1A). The histologic sections of the nodule revealed solid arborizing bands of oval to spindle cells that permeate in and around thyroid follicles without desmoplastic reaction (Figure 2A-2C). The tumor cells were associated with lymphoplasmacytic infiltrate and scattered psammoma bodies, in a background of lymphocytic thyroiditis. There were no classic patterns of papillary thyroid carcinoma or follicular growth. The tumor did not show any aggressive features such as extrathyroidal extension, elevated mitotic rate, or necrosis. Resection margins were negative for carcinoma. The right paratracheal lymph node was positive for metastatic carcinoma positive for TTF-1 with similar morphology as the thyroid carcinoma (Figure 2D) and one perithyroidal lymph node was negative for carcinoma.

**Figure 3:**
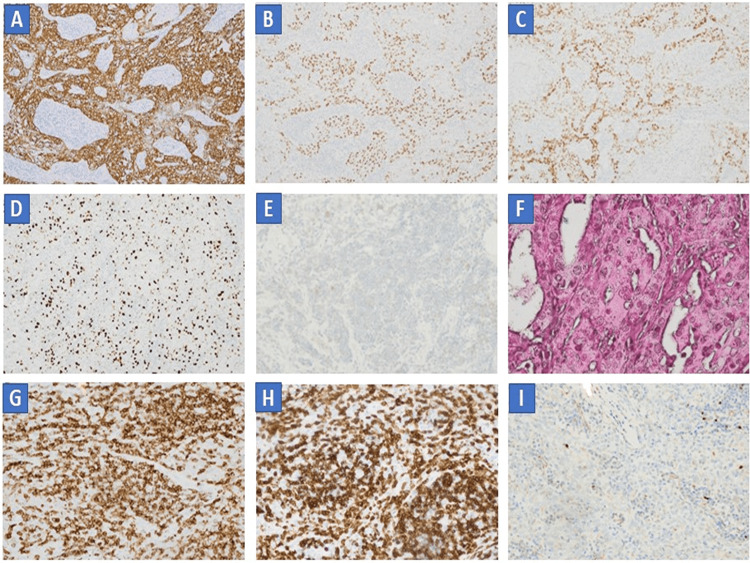
Immunohistochemistry (IHC) AE1/AE3 positive(A), P40 positive (B), TTF1 positive (C), Ki 67 proliferation index 5% (D), Calcitonin negative (E), EBER ISH negative (F), CD5 positive (G), CD3 negative (H), PAX-5 negative (I).

Though FNAC showed papillary architecture, similar morphology was not seen in the resection specimen even after an extensive search. Immunohistochemical stains performed showed that the tumor cells were positive for TTF1, PAX-8, AE1/AE3 confirming origin from thyroid follicular epithelium (Figure 3A-3C). Calcitonin was negative ruling out medullary thyroid carcinoma. In situ hybridization for EBV-encoded RNA (EBER-ISH) was negative in the tumor cells (Figure 3F). The background lymphocytes consisted of predominantly T cells positive for CD3, CD5 and scant B cells positive for CD20, PAX-5 (Figure 3G-3I). Metastatic lymphoepithelioma-like carcinoma (LELC) from other sites was excluded as radiology did not reveal any other primary lesions. Due to the absence of aggressive features of the tumor, no adjuvant hormonal therapy, chemotherapy, or radiotherapy was given to the patient. At the time of the study, the patient had no tumor recurrence on 15-month follow-up.

## Discussion

Lymphoepithelial carcinoma is the most common primary tumor of the nasopharynx [6]. It is characterized by syncytial arrangement of cohesive cells with indistinct cell margins and prominent non-neoplastic lymphoplasmacytic infiltrate. Epstein-Barr virus is usually integrated in these nasopharyngeal tumors and can be detected by in-situ hybridization. LELC could occur in other organs like lung [7], salivary gland [8], uterine cervix [9], and stomach [5], but its association with EBV infection is inconsistent. Though rare cases of LELC in thyroid have been reported it is not included in the WHO classification of thyroid cancers. LELC in the thyroid gland constitutes less than 1% of all thyroid carcinoma. It has been previously termed intrathyroidal epithelial thymoma, primary thyroid thymoma or carcinoma of the thyroid showing thymus-like differentiation [10]. LELC is a subtype of thyroid carcinoma and shows morphology similar to undifferentiated carcinoma of nasopharynx and LELC of other sites [11].

Shek et al. reported a lymphoepithelioma-like carcinoma of the thyroid gland in a 39-year-old female with a lack of association with EBV virus [10]. She presented with a right lower pole 2.4 cm (about 0.94 in) solid nodule with cystic component. Microscopically the tumor was well encapsulated with nests of cohesive syncytial appearing cells in a slightly fibrotic stroma heavily infiltrated by lymphocytes and occasional plasma cells. The tumor cells were large with vesicular nuclei, prominent eosinophilic nucleoli, and rare mitosis and were negative for thyroglobulin and calcitonin. The lymphocytes showed an equal number of B and T cells. EBV in situ hybridization was negative. Extensive investigations including a biopsy of nasopharynx failed to show another primary malignancy.

Huang et al. [9] reported an EBV-associated LELC with co-existing classic PTC in a 44-year-old female. She presented with a 4 cm left thyroid nodule and bilateral neck lymphadenopathy. Histology of the left lobe nodule revealed an undifferentiated carcinoma with lymphoepithelioma-like features with focal hyaline matrix and infiltration of lymphocytes. The tumor cells were negative for thyroid markers TTF-1 and PAX-8. EBER ISH was diffusely positive. Left neck lymph nodes were positive for metastatic carcinoma with lymphoepithelioma-like features and right neck lymph nodes were positive for metastatic papillary thyroid carcinoma, classic type. The right and left lobes of thyroid were extensively sampled but did not show classic-type PTC. The tumor was not completely resected due to laryngeal invasion.

Differential diagnoses to be considered for LELC of thyroid include intrathyroid thymic carcinoma (ITC), medullary thyroid carcinoma (MTC), metastatic LELC, anaplastic thyroid carcinomas, and subtypes of PTC. ITC shows fibrous bands separating islands or nests of squamoid or syncytial appearing tumor cells with occasional single-cell keratinization. The epithelial cells are positive for high molecular weight keratin, p63, p40, polyclonal pax8, CD5, CD117, and are negative for thyroid follicular markers. Stromal lymphocytic infiltration is one of the characteristic features of ITC and the lymphocytes are positive for CD3, CD5, and CD20 is variably positive [12]. MTC exhibits dispersed, pleomorphic cells with plasmacytoid, polygonal or spindle cell morphology, granular cytoplasm, and salt and pepper chromatin. Amyloid deposits are often observed in the background, and the tumor cells are usually positive for calcitonin and carcinoembryonic antigen (CEA), in the absence of thyroid follicular markers (thyroglobulin, TTF1) [13].

Other primary thyroid tumors to be considered include subtypes of PTC-like spindle cells and Warthin-like types. Thyroid spindle cell lesions are rare and spindle cell metaplasia in PTC is even rarer. Spindle cell areas may be focal or diffuse in PTC, but spindle cell subtype of PTC is characterized by a predominance (>50%) of spindle cells with nuclear features of PTC but they lack lymphoplasmacytic infiltrate. The presence of spindle cells does not modify the prognosis of the tumor. Spindle cell areas are made up of cytologically bland elongated cells, arranged in bundles, with less pronounced nuclear clearing or pseudo inclusions than classic PTC. Spindle cells do not show increased mitotic activity and necrosis. They are immunoreactive with pan-cytokeratin and markers of thyroid lineage (thyroglobulin, TTF1 and PAX8) but negative with calcitonin/chromogranin, confirming their follicular origin. These cells are not associated with post-fine needle aspiration reactive change. Warthin-like PTC can present as a well-circumscribed or infiltrative tumor and share morphologic similarities with Warthin tumor of the salivary gland. It is composed of papillae lined by oncocytic cells with the papillary cores rich in lymphocytes and plasma cells [4,5].

In our case, metastatic LELC was ruled out as radiology did not reveal any other primary lesions. Anaplastic thyroid carcinoma was excluded due to the expression of thyroid-specific markers, absence of aggressive features like extrathyroidal extension, marked nuclear pleomorphism, increased mitosis, and necrosis.

Classic PTC and other subtypes are best treated with surgery. Based on the tumor size and the presence of lymph node metastasis, surgeons may opt for lobectomy or total thyroidectomy with lymph node dissection. Radioactive iodine can be used to supplement thyroidectomy in patients with a tumor <2 cm (about 0.79 in) and distant metastasis or patients with a tumor >2 cm (about 0.79 in) and one of the following risk factors like gross extrathyroidal extension, age >45 years, lymph node and distant metastases [14]. The significance of association with EBV is not clear in the treatment of LELC of thyroid. Our patient did not receive any adjuvant treatment after total thyroidectomy and on 15 months follow-up, she is free of recurrent disease.

## Conclusions

We report a rare case of EBV-negative papillary thyroid carcinoma with lymphoepithelial features in a 29-year-old female patient. Since this case has lymphoepithelial morphology, microscopic features and immunohistochemistry must be done to rule out anaplastic differentiation and/or thymus differentiation, which will render an accurate diagnosis, as the prognosis for these cases is completely different. Furthermore, a thorough work-up to rule out metastasis from other sites, such as the lungs and bones, should be undertaken. A better understanding of the pathogenesis and progression of the disease would be useful in planning the treatment and follow-up, ultimately improving patient outcomes.

## Appendices

**Table 1:** Chart review

Case reference	Age and sex	Radiological findings	FNAC and molecular studies	Histopathology of the excised tumor	Immunohistochemistry	Follow up
Liu and Huang [11]	44-year-old female	A 4.0 × 3.0 × 1.7 cm hypodense mass with ill-defined margins in the left thyroid gland.	Loosely cohesive clusters of follicular epithelial cells, as well as an abundant dense extracellular matrix with few atypical epithelioid cells. No molecular was done.	Undifferentiated carcinoma with lymphoepithelioma-like features.	The tumor cells were positive for cytokeratin (AE1/AE3) and p40 but negative for TTF-1, Pax-8, and CD5. EBER-ISH was diffusely positive.	Patient was alive for 1 year and 10 months.
Shek et al. [10]	39-year-old female	A well-defined nodule 19 mm diameter over the lower pole.	Patient declined FNAC. No molecular was done.	A lobulated, well-encapsulated tumor that was characterized by nests of cohesive syncytial-appearing cells in a slightly fibrotic stroma heavily infiltrated by small lymphocytes and occasional plasma cells. The tumor cells were large with big vesicular nuclei and a prominent eosinophilic nucleolus, but mitotic figures were rarely detected.	The tumor cells were positive for MAK-6, but negative for thyroglobulin or calcitonin. Roughly equal number of T and B-cells as well as scattered S-100 positive Langerhans' cells was found in the lymphoid background. EBER was negative.	Patient was alive and free of tumor for 15 months follow-up.
Loh et al. [15]	28-year-old female	2.8×2.1×1.9 cm suspicion nodule in the right lower lobe of the thyroid gland	FNAC: papillary carcinoma of the thyroid gland (Bethesda system VI) Molecular negative for BRAF gene test	There are squamoid tumor cell nests in association with clusters of lymphocytes. There were findings of capsular invasion, but there was no lymphatic and vascular infiltration.	showed p63 positive and negative for CD5 and Bcl-2. EBV antibody test was performed and the results were negative (IgM and IgG)	Patient was alive and free of tumor for 32 months follow-up
Our case	29-year-old female	A 1.5 cm hypoechoic solid nodule with ill-defined margins.	Follicular cells with nuclear enlargement, nuclear overlapping, focal three-dimensional clusters with papillary. Architecture, few psammoma bodies and mixed population of lymphocytes. Molecular study showed gene expression alterations associated with thyroid cancer.	Solid arborizing bands of oval to spindle cells that permeate in and around thyroid follicles without desmoplastic reaction (Figure 2A-2C). The tumor cells were associated with lymphoplasmacytic infiltrate and scattered psammoma bodies.	Tumor cells were positive for TTF1, PAX-8, AE1/AE3 Calcitonin was negative. The background lymphocytes consisted of predominantly T cells positive for CD3, CD5 and scant B cells positive for CD20, PAX-5. EBER-ISH was negative.	Patient was alive and free of tumor for 15 months follow-up.
